# The biogenesis of β-lactamase enzymes

**DOI:** 10.1099/mic.0.001217

**Published:** 2022-08-09

**Authors:** Nikol Kaderabkova, Manasa Bharathwaj, R. Christopher D. Furniss, Diego Gonzalez, Tracy Palmer, Despoina A.I. Mavridou

**Affiliations:** ^1^​ Department of Molecular Biosciences, The University of Texas at Austin, Austin, Texas, USA; ^2^​ Centre to Impact AMR, Biomedicine Discovery Institute and Department of Microbiology, Monash University, Melbourne, Victoria, Australia; ^3^​ Science for Life Laboratory, Department of Molecular Biosciences, The Wenner-Gren Institute, Stockholm University, Stockholm, Sweden; ^4^​ Laboratoire de Microbiologie, Institut de Biologie, Université de Neuchâtel, Neuchâtel, 2000, Switzerland; ^5^​ Microbes in Health and Disease, Newcastle University Biosciences Institute, Newcastle University, Newcastle upon Tyne, UK; ^6^​ John Ring LaMontagne Center for Infectious Diseases, The University of Texas at Austin, Austin, Texas, USA

**Keywords:** antibiotics, antimicrobial resistance, β-lactamase, protein folding, protein translocation

## Abstract

The discovery of penicillin by Alexander Fleming marked a new era for modern medicine, allowing not only the treatment of infectious diseases, but also the safe performance of life-saving interventions, like surgery and chemotherapy. Unfortunately, resistance against penicillin, as well as more complex β-lactam antibiotics, has rapidly emerged since the introduction of these drugs in the clinic, and is largely driven by a single type of extra-cytoplasmic proteins, hydrolytic enzymes called β-lactamases. While the structures, biochemistry and epidemiology of these resistance determinants have been extensively characterized, their biogenesis, a complex process including multiple steps and involving several fundamental biochemical pathways, is rarely discussed. In this review, we provide a comprehensive overview of the journey of β-lactamases, from the moment they exit the ribosomal channel until they reach their final cellular destination as folded and active enzymes.

## Introduction

From the treatment of community acquired infections and chronic diseases to the performance of surgery, β-lactam antibiotics have played an essential role in the advancement of modern medicine and remain, to date, the most prescribed antibiotic drugs in the world, thanks to their low toxicity and their wide spectrum of activity against both Gram-positive and Gram-negative bacteria [1]. These antibiotics contain a key four-membered β-lactam ring through which they competitively inhibit the extra-cytoplasmic penicillin binding proteins (PBPs) that are responsible for the final cross-linking step during bacterial cell wall synthesis [2, 3]. Hindering the maturation of the cell wall, in turn, results in loss of cellular integrity and bacterial cell death.

The inactivation of β-lactam drugs by many bacterial species often leads to treatment failure. The most prevalent resistance mechanism against β-lactam compounds is the hydrolysis of their central β-lactam ring by enzymes called β-lactamases. These resistance determinants are found in both Gram-positive (secreted into the extracellular space or embedded into the membrane) and Gram-negative (translocated into the periplasmic space) species and, since they primarily serve as bacterial defence against molecules produced by other microorganisms, they predate the first clinical use of β-lactam antibiotics [4–6]. β-Lactamases are often encoded on plasmids and transposable elements and over six thousand enzymes [7] have now spread through the bacterial phylogeny. In addition to their broad dissemination, their capacity for functional diversification and inhibitor escape constantly complicates the development of agents designed to mitigate their activity [8].

### β-Lactamase classification

**Fig. 1.**
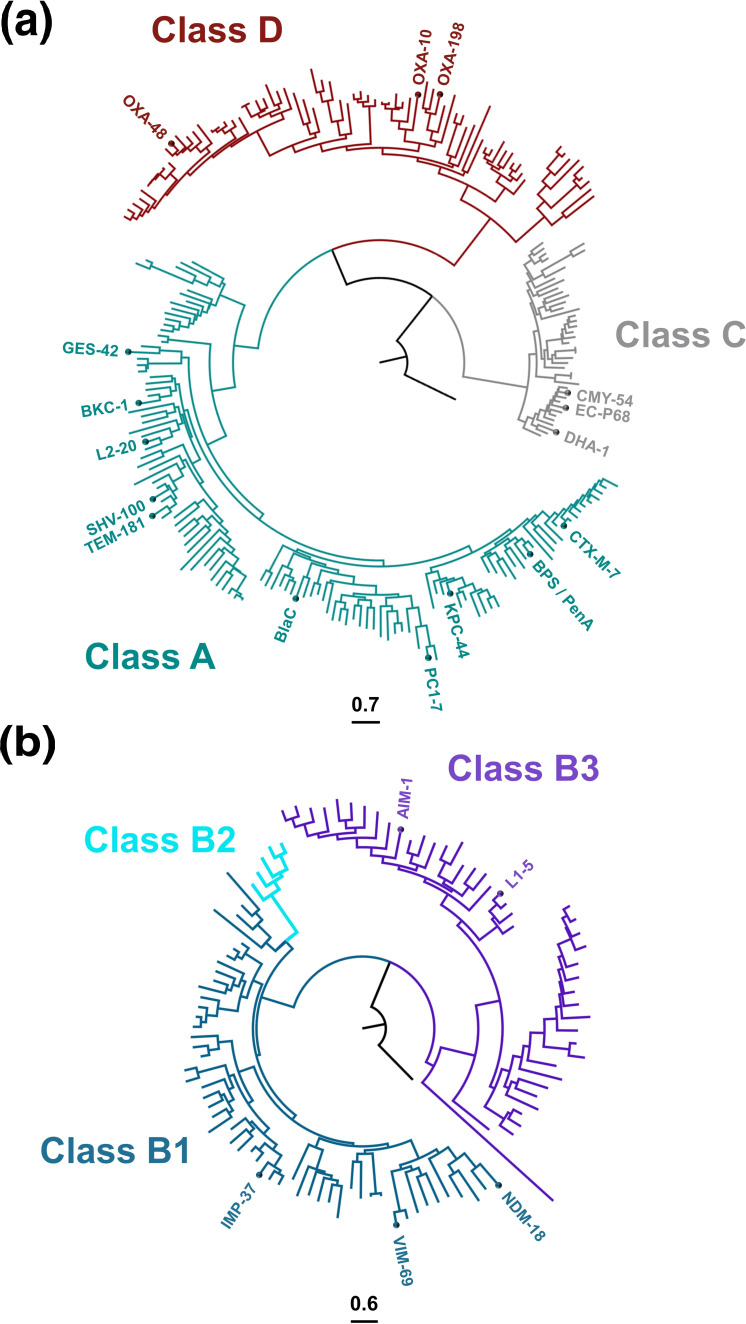
Phylogeny of β-lactamase enzymes. (**a**) Phylogenetic tree of β-lactamases from Ambler classes A, C and D, rooted on a d-alanyl-d-alanine carboxypeptidase enzyme from *Streptomyces* spp. (UniProt: DAC_STRSR [283]). (**b**) Phylogenetic tree of Class B β-lactamases rooted on the *E. coli* hydroxyacylglutathione hydrolase GloB (UniProt: GLO2_ECOLI [284]); sub-classes B1, B2 and B3 are indicated. Representative enzyme members of each phylogenetic group that is either discussed in the text or included in Table 1 are labelled and indicated by a circle. The trees were constructed as follows: all available protein sequences of β-lactamases were downloaded from http://www.bldb.eu [41]. Sequences were split in two groups (one group containing enzymes from classes A, C and D and a second group comprising enzymes from class B). Sequences for each group were then clustered using the *cd-hit* 4.8.1 software with an 0.8 identity threshold, and for clusters containing more than two sequences, one sequence per cluster was retained. All retained sequences were aligned using *muscle* 3.8.31 [285] and a phylogenetic tree was built using *IQ-TREE* 2.1.1 [286] with 1000 iterations; best-fit model was determined by *IQ-TREE* automatically.

**Fig. 2.**
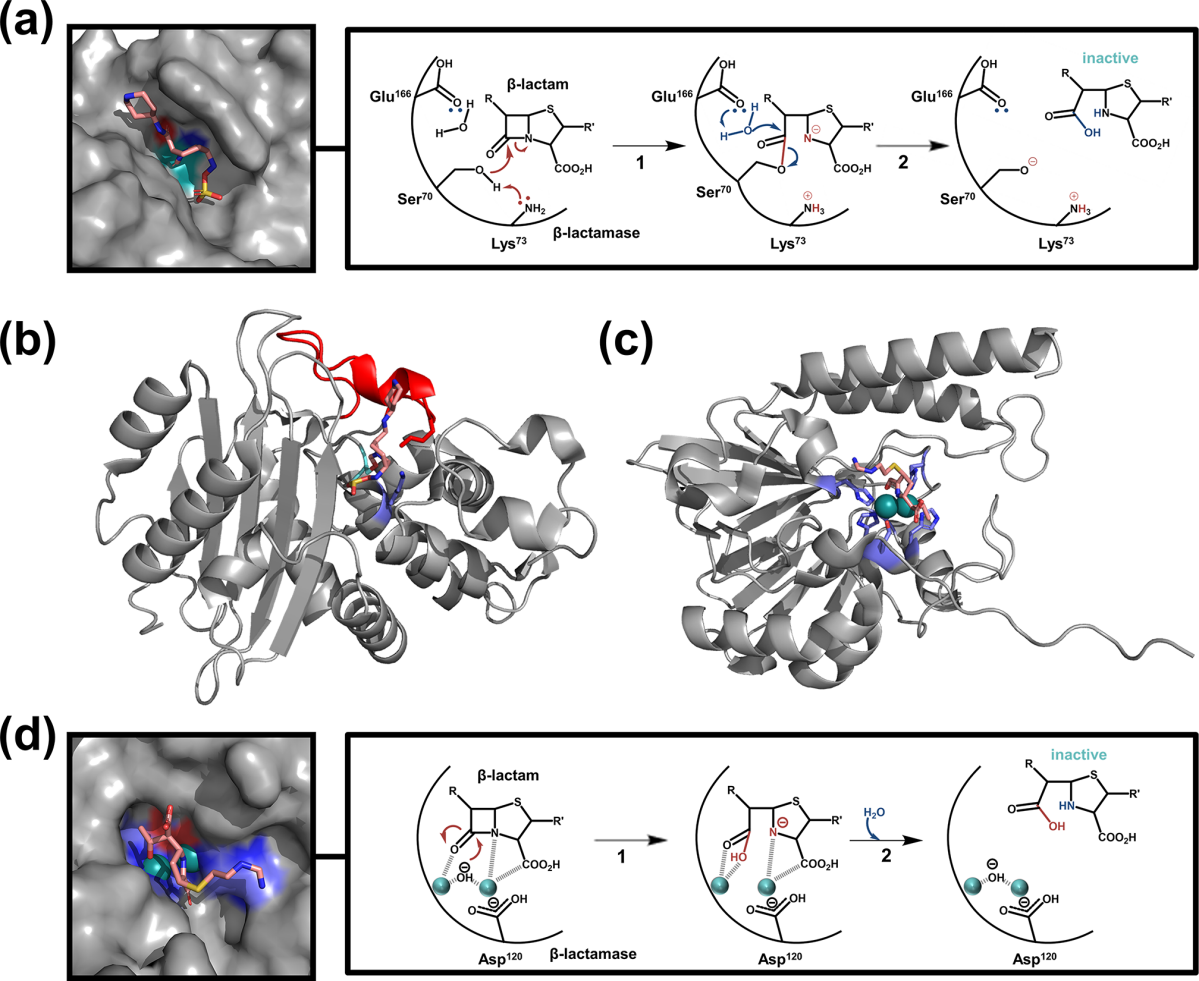
Structures and catalytic mechanisms of β-lactamase enzymes. (**a**) (left box) Rendering of the catalytic pocket representative of serine β-lactamase enzymes (KPC-2 is shown, PDB: 6QW9) in complex with a substrate (the new-generation β-lactamase inhibitor relebactam) in pink. Active-site residues playing an important role in β-lactam hydrolysis are indicated (Ser70 in cyan, Lys73 in blue, and Glu166 in red). (right box) Hydrolytic mechanism of serine β-lactamases based on the KPC-2 enzyme. Hydrolysis of β-lactam compounds happens in two steps, starting with acylation of the substrate by Ser70, which is first deprotonated by the nearby Lys73 (step 1) [236, 287, 288]. A de-acylation step occurring through a coordinated water molecule, which is first deprotonated by Glu166, follows (step 2), resulting in the release of the inactive β-lactam and the active form of the β-lactamase [236]. (**b**) Structural representation of serine β-lactamase enzymes; as in panel (**a**), KPC-2 in complex with relebactam (in pink) is shown (PDB: 6QW9). Active-site residues playing an important role in β-lactam hydrolysis are indicated in stick representation (Ser70 in cyan, Lys73 in blue, and Glu166 in red), while the Ω-loop residues are coloured in red. (**c**) Structural representation of class B1 and B3 metallo-β-lactamase enzymes (L1-1 is shown, PDB: 6UAF) in complex with a substrate (imipenem) in pink. Class B1 and B3 enzymes generally require two Zn(II) ions (cyan spheres) for activity. These metal centres are ligated by histidine (His116, His118, His121, His196, and His263 for L1-1; in blue) and aspartic acid (Asp120 for L1-1; in red) residues [9, 83–85, 289, 290] (**d**) (left box) Rendering of the catalytic pocket representative of class B1 and B3 metallo-β-lactamase enzymes. As in panel (**c**), L1-1 is shown (PDB: 6UAF) in complex with imipenem (in pink); active-site Zn(II) ions are represented by cyan spheres and ligating residues are coloured blue (histidines) or red (aspartic acid). (right box) Hydrolytic mechanism of B1 and B3 metallo-β-lactamases based on the L1-1 enzyme (the metal-ligating histidine residues are omitted for clarity). The active-site Zn(II) ions coordinate the β-lactam in the catalytic pocket [9, 83–85, 289, 290], and direct the nucleophilic attack of a stabilised hydroxide ion (step 1). Subsequent proton transfer leads to β-lactam hydrolysis and release of the active metallo-β-lactamase (step 2) [234, 289–291]. All structures presented in this figure were rendered using PyMol (Version 2.3.5, Schrodinger, LLC).

β-Lactamases are most commonly classified based on their amino acid sequence similarity using the Ambler classification system that encompasses classes A-D [1, 9]. Enzymes belonging to different classes have evolved independently, and each class contains numerous phylogenetically distinct enzyme groups (Fig. 1). Classes A, C, and D (Fig. 1a) comprise serine-based β-lactamases, whose activity relies on a conserved serine residue in their active site, and which are characterised by notable structural similarity to the PBPs [1, 10]. Their evolution from the membrane-anchored PBPs likely occurred through the development of their ability to perform a diacylation step [11–13] that allows the release of the inactive β-lactam substrate from the catalytic pocket, and through, in many cases, the loss of their membrane anchor [11]. By contrast, class B lactamases (Fig. 1b) have evolved along a different track from metallo-hydrolase enzymes and perform their function through one or two catalytically active Zn(II) ions in their active site [1, 3, 14, 15]. Structural differences between serine β-lactamases (class A, C and D) and metallo-β-lactamases (class B) result in distinct hydrolytic mechanisms (Fig. 2) that influence the activity spectra of these enzymes, as well as their interaction with β-lactamase inhibitor compounds, developed to attenuate their function and potentiate β-lactam antibiotics.

In addition to the Ambler system, β-lactamases can also be classified according to their hydrolytic activities into narrow-spectrum enzymes, extended-spectrum β-lactamases (ESBLs), and carbapenemases. Narrow-spectrum β-lactamases can only break down penicillins and early (first- and second-) generation cephalosporins, while ESBLs act on most β-lactam antibiotics, except for last-resort carbapenem drugs. Thanks to this broad range of activities some organisms possess combinations of enzymes with complementary hydrolytic spectra, thus causing infections that are extremely challenging to treat with clinically available β-lactams. This problem is routinely reported for the opportunistic pathogen *Stenotrophomonas maltophilia *, found in chronic cystic fibrosis infections. Co-evolution of the chromosomally-encoded class A ESBL L2 and the class B carbapenemase L1 enzymes in this species results in resistance to all β-lactam antibiotics [16–19]. Another way to classify β-lactamases according to their hydrolytic spectra is by following the Bush-Jacoby-Medeiros system. This classification method takes into consideration not only the hydrolytic activity of each enzyme, but also its structure and interaction with inhibitor compounds, therefore generating a more informative, albeit complicated, enzyme classes [20, 21]. As the Ambler system is the simplest and most widely used method to classify β-lactamases, we will use this nomenclature throughout this review article.

### Class A β-lactamases

Class A is the most diverse subgroup of the Ambler classification system [7] and contains some of the most clinically concerning β-lactamases, including TEM and SHV enzymes (* Escherichia coli* and *Klebsiella pneumoniae *, respectively), along with GES (* Pseudomonas* spp., Enterobacteriaceae [22–25]), KPC (* K. pneumoniae* [26]), and CTX-M enzymes (Enterobacteriaceae [27]) (Table 1). Several class A β-lactamases are also found in Gram-positive bacteria, for example BCL-1 (* Bacillus* spp. [28]), BlaC/BlaS (* Mycobacterium* spp. [29, 30]), and BlaL/BlaU (* Streptomyces* spp. [31]). In Class A enzymes their active site is sandwiched between an α-helical domain and an α/β domain (Fig. 2b). Their hydrolytic activity varies from narrow-spectrum to carbapenem-hydrolysing enzymes and depends on a two-step process involving the catalytic Ser70 residue (as defined in the consensus amino acid numbering scheme for Class A enzymes [32]), a coordinated water molecule, as well as lysine and glutamic acid residues that are part of the active site (see Fig. 2a for a detailed description of β-lactam hydrolysis by serine β-lactamases like Class A enzymes). The catalytic pocket in proteins from this class is framed by an Ω-loop (Fig. 2b; shown in red), a non-regular unit of secondary structure which traces a loop-shaped path in three-dimensional space and is found at the surface of many globular proteins [33, 34]. In β-lactamases in particular, the Ω-loop contains a catalytic glutamic acid that is linked to its neighbouring residue by a highly conserved *cis* peptide bond [35–37]. The activity of many class A β-lactamases can be abrogated using classical inhibitor compounds, such as clavulanic acid, tazobactam or sulbactam. Despite this, several enzymes from this class, like the highly evolved and disseminated KPC carbapenemases, evade such inhibitors and are currently an imminent threat to the longevity of β-lactam antibiotics [8, 38].

**Table 1.** Overview of β-lactamase enzymes that have been extensively studied, most of which are clinically significant. The ‘activity’ column refers to their hydrolytic spectra, with narrow-spectrum enzymes only breaking down penicillin and early cephalosporin compounds and extended-spectrum β-lactamases (ESBLs) and carbapenemases processing complex β-lactam drugs such as third/fourth-generation cephalosporins and carbapenems, respectively. The ‘inhibition’ column refers to either classical inhibitor susceptibility i.e. susceptibility to inhibition by clavulanic acid, tazobactam or sulbactam, or inhibition by new-generation compounds like avibactam, vaborbactam and relebactam [8]; the * denotes that only some members of the particular phylogenetic family are susceptible to inhibition even by these newer compounds. The ‘mobile’ column refers to the genetic location of the β-lactamase gene; ‘yes’ indicates that the gene of interest is located on a plasmid or mobile element, while ‘no’ refers to chromosomally-encoded enzymes. As seen in the ‘export’ column, the majority of enzymes are translocated through the Sec system, including β-lactamases that have a ‘lipobox’ (denoted ‘lipo’), which eventually localize in the membrane through acylation of their N-terminal cysteine [247, 292]. Finally, the ‘organism’ column refers to the bacterial species that most commonly express the β-lactamase enzymes in question

β-lactamase	Class	Activity	Inhibition	Mobile	Export	Organisms	Source
BPS/PenA	A	ESBL	classical	no	Sec (lipo)	* B. pseudomallei *	[293]
BKC-1	A	carbapenemase	classical	yes	Sec/Tat	* K. pneumoniae *	[178, 209]
BLAC	A	narrow-spectrum	new generation*	no	Tat	* M. tuberculosis *	[29, 179, 234]
CTX-M	A	ESBL	tazobactam	yes	Sec	Enterobacteriaceae	[27, 103]
GES	A	ESBL/ carbapenemase	classical	yes	Sec	Enterobacteriaceae, * P. aeruginosa *	[22, 114]
KPC	A	ESBL/ carbapenemase	new generation*	yes	Sec	* K. pneumoniae *	[38, 104, 294, 295]
L2	A	ESBL	classical	no	Tat	* S. maltophilia *	[8, 177]
PC1	A	narrow-spectrum	classical	no	Sec (lipo)	* S. aureus *, *Bacilli *	[144, 145]
TEM	A	narrow-spectrum/ESBL	classical	yes	Sec	Enterobacteriaceae	[296, 297]
SHV	A	narrow-spectrum/ESBL	classical	yes	Sec	* K. pneumoniae *	[298, 299]
IMP	B1	carbapenemase	–	yes	Sec	* P. aeruginosa *, *K. pneumoniae,* * A. baumannii *	[73–75]
NDM	B1	carbapenemase	–	yes	Sec (lipo)	Enterobacteriaceae	[69, 279]
VIM	B1	carbapenemase	–	yes	Sec	* P. aeruginosa *, Enterobacteriaceae	[71, 72]
AIM	B3	carbapenemase	–	yes	Sec	* P. aeruginosa *	[78, 79]
L1	B3	carbapenemase	–	no	Sec	* S. maltophilia *	[86, 177]
AmpC/EC	C	narrow-spectrum/ESBL	new generation*	yes	Sec	Enterobacteriaceae	[300–303]
DHA-1	C	ESBL	classical	yes	Sec	*M. morganii*, * S. enteritidis *, other Enterobacteriaceae	[47–50]
CMY	C	ESBL	new generation*	yes	Sec	*K. pneumoniae,* * E. coli *	[42, 43]
OXA-10	D	ESBL	new generation*	yes	Sec	* P. aeruginosa *	[109, 304]
OXA-48	D	carbapenemase	new generation*	yes	Sec	* K. pneumoniae *	[8, 56, 57, 305]
OXA-198	D	carbapenemase	new generation*	no	Sec	* P. aeruginosa *	[306]

ESBL, extended-spectrum β-lactamase; Sec system, general secretion system; Tat, twin arginine translocation.

### Class C serine β-lactamases

Class C cephalosporinases are the group with the most members [7] and, due to their near indistinguishable sequences in comparison to the native PBPs, their identification and naming are not straightforward [39, 40]. The most commonly discussed β-lactamases from this class are the AmpC enzymes from Enterobacteriaceae, sometimes also named EC enzymes [41]. Other class C β-lactamases are also widely disseminated across the chromosomes of bacterial pathogens such as *Citrobacter freundii* (CMY enzymes [42, 43]) or *Acinetobacter baumannii* (ADC enzymes [44]), and have been found on plasmids harboured by *K. pneumoniae* (ACT-1 [45, 46]) or *Salmonella enteritidis* (DHA-1 [47–50]) (Table 1). Their three-dimensional structures are similar to class A enzymes (Fig. 2a, b) except for their active site, which is more open and located at the edge of their central β-sheet [40, 51]. Due to this considerably larger catalytic pocket, their activity is less affected by classical inhibitor compounds [8, 51, 52]. That said, novel inhibitors, such as avibactam or the boronic acid vaborbactam, have been shown to be effective against enzymes of this class [8, 53, 54].

### Class D serine β-lactamases

Class D enzymes are distinct from other types of serine-β-lactamases, with less than 20 % sequence identity [8, 55, 56]. The most prevalent representatives of this class are the OXA family of mobile β-lactamases found in many Gram-negative pathogens; the inhibitor-resistant carbapenemase OXA-48, initially isolated from *K. pneumoniae *, is the most clinically challenging enzyme of this group (Table 1) [7, 56, 57]. With over 750 different enzymes, sub-divided into over 50 phylogenetic sub-families (Fig. 1a), OXAs exhibit a diverse range of hydrolytic profiles often originating from single amino acid variations. The shape of their active site and consequently the hydrolytic spectrum of these enzymes, is also affected by a highly defined Ω-loop harbouring a conserved disulfide bond [7, 55, 58, 59]. Their hydrolytic mechanism largely resembles that of other serine β-lactamases (Fig. 2a), although there are a few differences. Studies on the OXA-1 and OXA-10 enzymes have shown that the first acylation step requires the activation of Ser67 by a carboxylated Lys70, and the second de-acylation step is slowed down by poor water activation [60–63]. With only minor exceptions, such as OXA-18, these β-lactamases are not inhibited by available compounds [52, 64]. In addition, although until recently class D β-lactamases were believed to be exclusive to Gram-negative species, examples of enzymes from this class have now been found in Gram-positive *Bacillus* spp. (BAT-1, BPU-1, BSU-1 [65]) and in *Clostridium difficile* (CDD-1 [66]).

### Class B metallo-β-lactamases

While metallo-β-lactamases form the smallest and least diverse class, this group contains highly active broad-spectrum enzymes that are responsible for antibiotic resistance in some of the most serious nosocomial chronic infections caused by both Gram-positive and Gram-negative pathogens [67]. They can be sub-divided into three distinct groups (Fig. 1b), **B1** (NDM [68, 69], BcII [70], VIM [71, 72], IMP [73–75]), **B2** (CphA [76], SFH [77]), and **B3** (AIM [78, 79], GOB-18 [80, 81], L1 (16–19)), based on their sequence similarity and zinc-binding characteristics (Table 1). Structurally, all metallo-β-lactamases have a common αββα fold with the active site located at the edge of the central β-sheets (Fig. 2c [82]). B1 and B3 enzymes require two Zn(II) ions for activity, and these metal centres are ligated by conserved histidines and an aspartic acid residue, (see Fig. 2d for a detailed description of β-lactam hydrolysis by B1/B3 metallo-β-lactamases). Conversely, B2 enzymes only contain a single Zn(II) ion in their active site, along with a conserved catalytic lysine (Lys224), and binding of a second Zn(II) inhibits their function [9, 83–86]. Due to their unique and divergent hydrolytic mechanism that depends on the use of Zn(II) ions rather than a serine residue, there are currently no clinically available β-lactamase inhibitors for metallo-β-lactamases, something that is cause for serious concern [3, 8].

### Acquisition of β-lactamase enzymes

Most clinically important β-lactamase enzymes that can break down last-generation β-lactams are found on mobile genetic elements. Nonetheless, even these ‘advanced’ β-lactamases originate from narrow-spectrum chromosomally-resident enzymes that were acquired by pathogens through horizontal gene transfer and have then mutated into broader-spectrum hydrolases. Examples of such archetypical chromosomally-resident β-lactamases include the enzymes of the SHV family (* K. pneumoniae *) [87, 88] that can hydrolyse penicillin and ampicillin or AmpC (* Pseudomonas aeruginosa *) that breaks down early-generation cephalosporins [89, 90]. Alarmingly, phylogenetic analysis suggests that β-lactamase transfer does not solely occur within the Gram-negative or Gram-positive polyphyletic groups. The Gram-negative ROB-1 enzyme from *Haemophilus influenzae* and AC1-1 from *Acidaminococcus fermentans* have also been found in numerous Gram-positive species [91, 92].

Mobilisation of β-lactamase genes occurs through plasmids, transposons, insertion sequences [87, 93, 94] or integrons [95, 96] that are disseminated through bacterial populations via conjugation, transformation or transduction. Conjugative plasmids play a critical role in the spread of antibiotic resistance determinants and multiple β-lactamases have been found encoded on a single plasmid. Recently, the metallo-β-lactamase VIM-1 has been shown to be disseminated on a Inc*A/C1* plasmid, which also carries the ESBL SHV-12, in *K. pneumoniae* [97], while the Inc*F* plasmids, encoding TEM-1 and OXA-1, often acquire an additional CTX-M enzyme, such as CTX-M-15, in *E. coli* ST131 [98, 99]. Other types of plasmids contributing to the spread of β-lactamases include, but are not limited to, Inc*I* plasmids carrying CTX-M-55 or KPC-3 in *E. coli* and *K. pneumoniae* [100–102], Inc*K* plasmids carrying CMY-2 in *E. coli* [103], and Inc*X* plasmids carrying KPC-3 or NDM-7 in *E. coli* and *C. freundii* [104–107]. Transposon-dependent transfer has also been observed for β-lactamases. For example, the narrow-spectrum inhibitor-resistant TEM-67, which is encoded on the pANG-1 plasmid in *Proteus mirabilis *, can be incorporated into the *E. coli* chromosome through the Tn*1* transposon and IS*26* insertion sequences [108]. Similarly, the ESBLs CTX-M-14 and CTX-M-15, originating from a resident β-lactamase found in *Kluyvera* spp. [109, 110], have been shown to mobilise and insert into the chromosome of *K. pneumoniae* and other Enterobacteriaceae via the IS*Ecp1* or IS*CR1* elements [107, 111, 112]. The mobile carbapenemase KPC-2 is also often associated with transposons, and more specifically with Tn*4441* that is flanked by either IS*Kpn6* or IS*Kpn7* [113]. Notably, although class B3 enzymes were thought to be immobile, the gene for the recently characterised metallo-β-lactamase AIM-1 from *P. aeruginosa* [78, 79] was shown to be flanked by IS*CR* elements that allow its mobilization into other strains [78, 79]. Finally, β-lactamases have been frequently associated with class I integrons, as seen with GES-1 from Enterobacteriaceae and other Gram-negative pathogens [114–118]. In these cases, their mobilization depends on the co-encoded integrase enzymes that catalyse their recombination into the chromosome through the flanking recombination sites, *att*I and *att*C [119].

For β-lactamase genes, transfer is especially promoted in environments where β-lactam-producing organisms co-exist with non-producing species [120]. Some of these horizontally acquired enzymes, encoded on plasmids, can ultimately be transferred back onto the chromosome of their new host, something that may offer more tightly regulated gene expression and could decrease the fitness costs associated with plasmid carriage and maintenance [112]. While plasmid-mediated β-lactamases, like ones belonging to the TEM, CTX-M and NDM families, have successfully evolved in various hosts, the expression of predominantly chromosomal species, such as SME-1 (* Serratia marcescens *) and SPM-1 (* P. aeruginosa *) impose a large fitness burden in *E. coli *, both in the presence or absence of antibiotics [121, 122]. These effects are likely caused by species-specific requirements for the biogenesis of these enzymes, some of which will be discussed in more detail in the following sections.

## Pre-translocation: the journey towards the cytoplasmic membrane

**Fig. 3.**
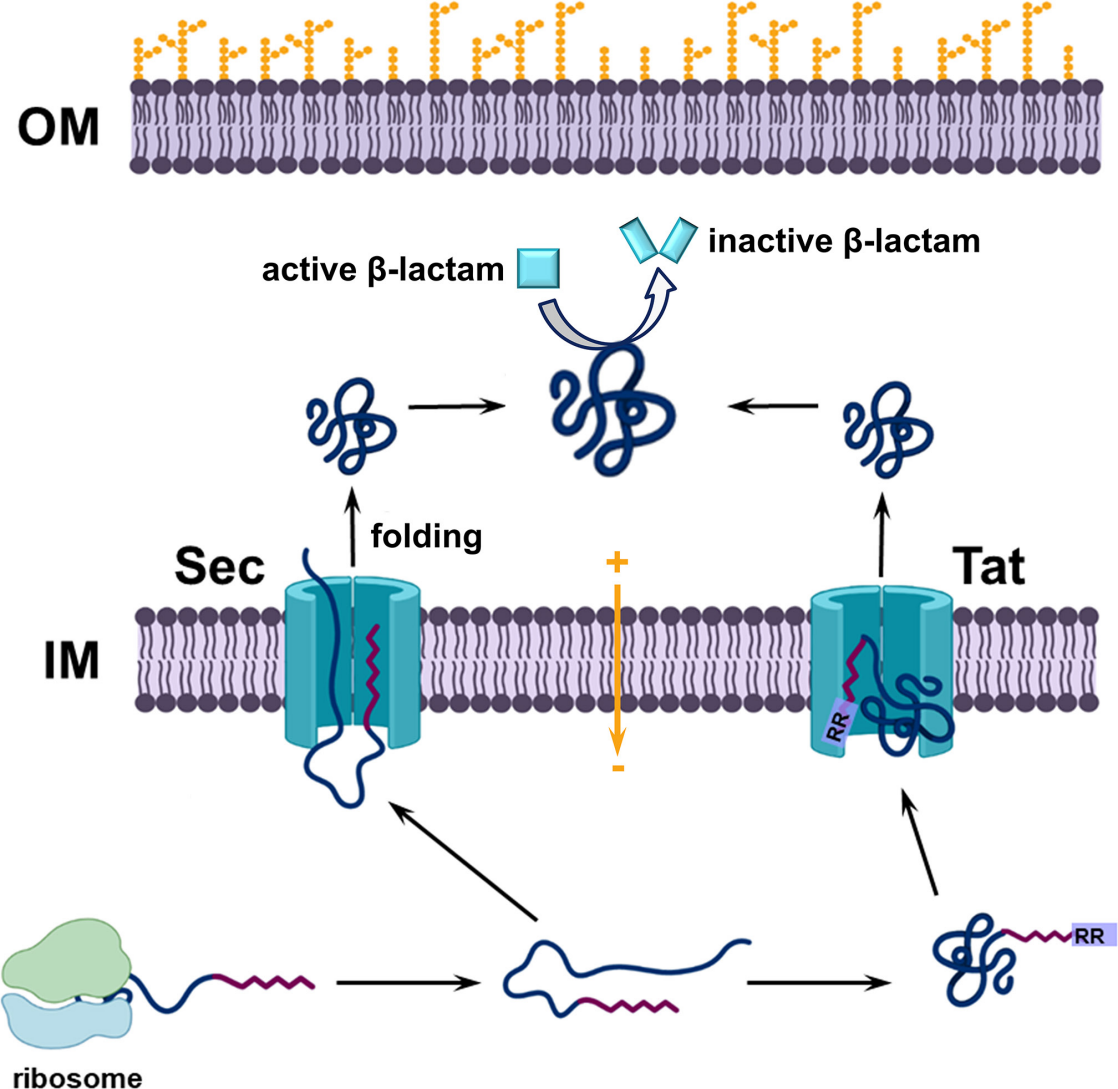
Overview of β-lactamase biogenesis. β-Lactamase enzymes acquire their native active form only after translocation through the cytoplasmic membrane and subsequent folding. After translation in the cytoplasm, the β-lactamase nascent chain reaches, with or without the help of chaperones (not shown), the Sec and Tat translocation pathways, which, in turn, help it cross the inner membrane barrier and fold on the other side. OM, outer membrane; IM, inner/cytoplasmic membrane.

β-Lactamase enzymes are synthesized in the cytoplasm as precursor molecules (pre-β-lactamases) carrying an N-terminal signal sequence. Specific elements within the signal sequence promote interaction with the general secretion (Sec) system or twin arginine translocation (Tat) pathways [123], through which pre-β-lactamases are exported to the periplasm (Gram-negative species) or secreted to the extra-cellular space (Gram-positive species); an overview of the biogenesis of β-lactamases is shown in Fig. 3.

**Fig. 4.**
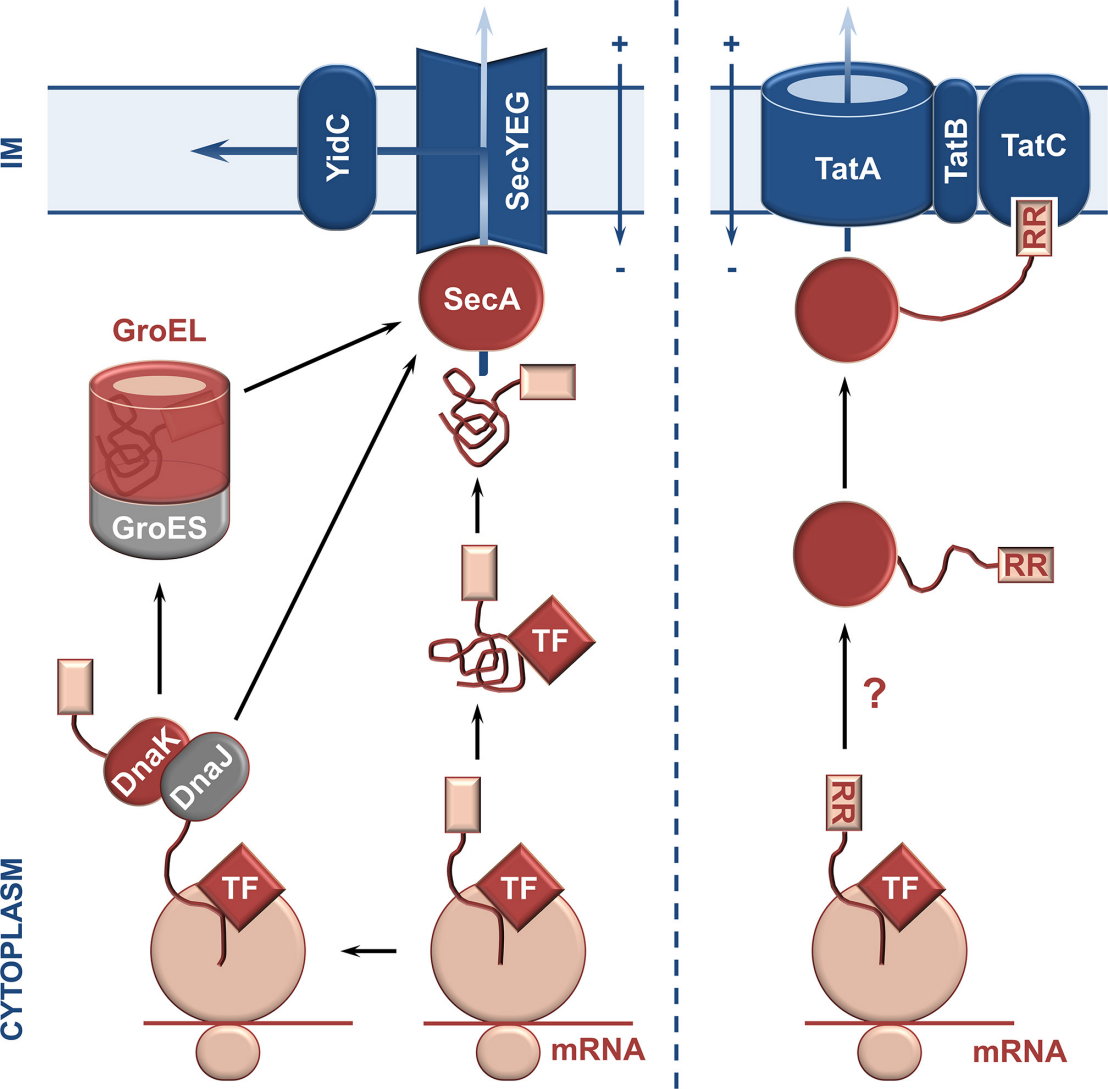
Overview of the cytoplasmic trajectory of β-lactamases. Enzymes translocated though the Sec system (left) interact, in their unfolded form, with trigger factor (TF), the DnaK-DnaJ-GrpE pathway, and/or GroEL/GroES before they are targeted, through their signal sequence (pink box), to the SecYEG channel. At that point, they are either translocated to the periplasm or, with the help of YidC [307], integrated into the inner membrane. There is no evidence to date that β-lactamases translocated through the Tat apparatus (right), i.e. enzymes with a twin-arginine (RR)-containing signal sequence, interact with general chaperones of the heat shock pathway. OM, outer membrane; IM, inner/cytoplasmic membrane.

Despite many pre-β-lactamases being well-known Sec substrates, their translocation is independent of the Sec-dedicated chaperone SecB [124, 125], and no other translocation-specific chaperones have been conclusively shown to specifically interact with pre-β-lactamases. It is known that some enzymes, such as TEM, cross the cytoplasmic membrane post-translationally [126, 127], which means that after release from the ribosome, misfolding due to non-native interactions can result in translocation-inactive forms and, in extreme cases, loss of hydrolytic activity or cellular toxicity [128–131]. This suggests that cytoplasmic chaperones may play a role in promoting translocation by influencing protein folding directly after the nascent pre-β-lactamase is released from the ribosome. Further, like with any other protein that requires oxidative folding in the periplasm, β-lactamase precursors with more than one cysteine residue are kept in their reduced thiol state by the cytoplasmic thioredoxin and glutaredoxin enzymes, including the thioredoxin reductase TrxA [132, 133]. Fig. 4 depicts an overview of the cytoplasmic trajectory of β-lactamases, starting with their release from the ribosome and finishing with their translocation into the cell envelope; several scenarios and multiple cytoplasmic chaperones are shown because, despite extensive study, the role of each chaperone is not yet fully understood due to a high level of functional redundancy [130, 134–139]. The formation of chaperones and translocation machinery components involved in the biogenesis of β-lactamases is not discussed in this review; for further information on these topics we refer the reader to the reviews by Santra *et al*. [140], Balchin *et al*. [141] or Jiang *et al*. [142].

### Trigger factor

Cytoplasmic pre-β-lactamase folding has been mostly studied using two enzymes, TEM, a Gram-negative β-lactamase from *E. coli* [143], and PC1, a Gram-positive enzyme from *Staphylococcus aureus* [144, 145]. For these proteins a stepwise, chaperone-driven folding model has been proposed, whereby the unfolded state of the protein is in exchange with several intermediate states, and the rate limiting step of the process is the transition between one of these transient intermediates and the final native fold [143, 144, 146–148]. Initial folding of the precursor polypeptide occurs co-translationally within the ribosomal exit channel, where spatial restrictions allow the formation of small α-helical domains [149, 150], as a result of the interactions between the amino acid side chains of the nascent polypeptide and the cytoplasm. Folding continues after the nascent polypeptide exits the ribosome through interaction with the ribosome-associated chaperone trigger factor (TF) [130, 150, 151]. This elongated protein has been shown to interact with the majority of nascent chains, including pre-β-lactamases [152]. These interactions offer steric protection to the partially folded polypeptides which may then interact further with the general chaperones of the heat shock pathway (σ^32^), namely DnaK-DnaJ-GrpE and GroEL-GroES, before eventually reaching the appropriate translocation apparatuses [135, 137, 151, 153–156]. It should be noted that any secondary structure acquired in the cytoplasm post-translationally is lost prior to translocation across the inner membrane through the Sec translocation system; conversely fully folded β-lactamases are translocated using the Tat translocation system.

### DnaK-DnaJ-GrpE

The majority of classic DnaK-DnaJ-GrpE substrates are proteins of limited solubility and low abundance that are usually part of heterooligomeric assemblies in the cytoplasm or the cytoplasmic membrane [138, 157–159]. Nonetheless, DnaK-DnaJ-GrpE have also been shown to play a role in the biogenesis of approximately 3 % of periplasmic proteins [159], including the Sec-dependent β-lactamase enzyme GOB-18 from *Elizabethkingia meningoseptica* [81]. GOB-18 is a class B3 metallo-β-lactamase with an unusual active form that contains only a single Zn(II) metal centre (usually B3 enzymes require two Zn(II) ions for activity); replacement of the conserved residues that coordinates the zinc, His116, and the nearby Ser221, with Gln and Met, respectively, leads to this enzyme acquiring carbapenemase activity [80, 81]. GOB-18 biogenesis shows minor dependence on TF, suggesting that the nascent chain is engaged by TF during or immediately after translation [81]. Transient interactions with the chaperones DjlA, DnaJ or CbpA through the recognition of its extended hydrophobic regions lead to the transfer of pre-GOB-18 to DnaK [81, 148]. This interaction is critical for the biogenesis of this β-lactamase, as proven by the fact that its absence results in the loss of β-lactamase hydrolytic activity in the periplasm [81, 148]. After delivery of pre-GOB-18 to the Sec system, the nucleotide exchange factor GrpE promotes the release of ADP from DnaK. ADP release allows the binding of a new ATP molecule to DnaK, which promotes substrate release and returns DnaK to a state in which it is competent to accept new substrates [81, 134, 160, 161]. Beyond studies on GOB-18, research to date suggests that the DnaK-DnaJ-GrpE pathway plays only a minor role in the biogenesis of β-lactamase enzymes [134].

### GroEL-GroES

While DnaK-DnaJ-GrpE appear to rarely be involved in β-lactamase biogenesis, there are numerous reports of the interaction between pre-β-lactamases that are translocated through the Sec system and the highly abundant chaperones GroEL-GroES; the β-lactamases that have been studied in this context are narrow-spectrum members of the TEM or AmpC families [129, 146, 147, 162]. The strong affinity of GroEL-GroES for the Sec signal sequence [163], and additional interactions with the exposed hydrophobic regions of the pre-β-lactamase enzymes [146, 147, 164], drive the transfer of the partially-folded precursors from TF or, occasionally, DnaK-DnaJ-GrpE to GroEL-GroES [130, 159]. It has been shown that the 1 : 1 interaction between pre-TEM-2 and the heptameric rings of GroEL removes any pre-existing secondary structure [146, 147, 164, 165]. Ultimately, this leads to localization of the pre-β-lactamase:GroEL-GroES complex at the membrane and interaction with SecA, thus initiating its translocation by the Sec system [125, 129, 162, 166, 167]. More recently, pre-TEM was identified as a Class I substrate of GroEL-ES in an unbiased proteomics screen independently confirming the earlier experimental results on β-lactamase-chaperone interactions [137]. Overall, the role of GroEL-GroES in this process is to establish, through its chaperone/unfolding activity, an equilibrium between folded and unfolded β-lactamase precursors that effectively keeps the pre-β-lactamases in a translocation-competent state prior to its transport across the inner membrane [137, 146, 162, 163, 168].

Neither GroEL-GroES nor the DnaK-DnaJ-GrpE pathway have been implicated in the biogenesis of Tat-translocated β-lactamases. Despite this, research on other Tat substrates shows that DnaK interacts productively with the signal sequences of the multicopper oxidase CueO [169], Tat-translocated GFP chimaera proteins, a truncated form of the trimethylamine N-oxide reductase (TorA502 [170]) and the catalytic subunit of the dimethyl sulfoxide reductase DmsA [159, 171]. In addition, in the absence of DnaK, GroEL-GroES also appears to interact with the Tat-dependent NiFe-hydrogenase 1 [172], a TorA-GFP chimaera [170], and AmiA [137, 169], although the existing evidence for these interactions is less reliable.

## Translocation: crossing the membrane barrier

Pre-β-lactamases are exported into the periplasm via one of two general systems, the Sec [173] or the Tat pathway [174] (Fig. 4). The majority of β-lactamases identified to date are transported across the cytoplasmic membrane in an unfolded state by the Sec system [173, 175], and following translocation, they depend on periplasmic folding pathways and other folding factors to achieve their final active conformation. By contrast, the few pre-β-lactamases that are translocated in their fully folded conformation via the Tat system, do not need periplasmic components, as they are functional immediately upon release from the Tat translocon (unless anchoring in the membrane is required) [174, 176].

Notably, the genetic locus of β-lactamase enzymes seems to correlate with their translocation process. In particular, plasmid-associated β-lactamases, such as AmpC, CTX-M-14, TEM-1 [177], and KPC-2 [178] are always translocated via the Sec system, but enzymes encoded on bacterial chromosomes, for example L2 (177), BlaC [179], and PenA [180], can also be exported through the Tat pathway. Therefore, it is worth keeping in mind that the origin of the β-lactamase could play a crucial role in signal sequence adaptability, along with other factors that affect the folding and translocation kinetics of these enzymes. Nonetheless, crossover is also possible during transfer from one host to another, for example, BlaC, an exclusively Tat-dependent β-lactamase in *Mycobacterium tuberculosis* [179] was shown to be translocated via the Sec translocon in *E. coli* [177].

### Signal sequence

**Fig. 5.**
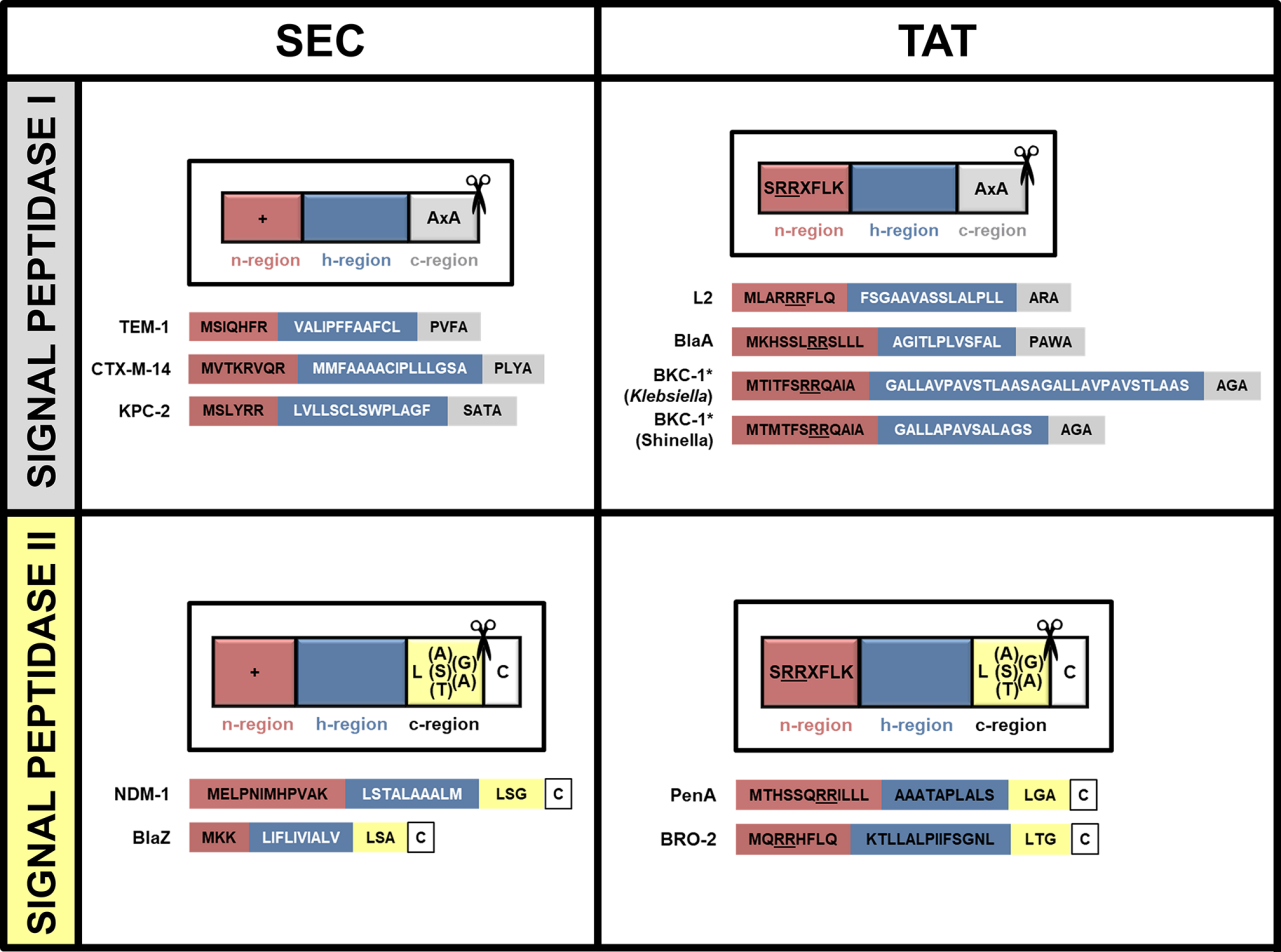
Signal sequences of Sec- and Tat-translocated β-lactamases. β-Lactamase signal sequences for enzymes known to be translocated through the Sec and Tat pathways are shown (single-letter amino acid code is used for simplicity), along with a schematic representation of the type of signal peptide for each case; the scissors indicate the position of the signal peptidase cleavage site. *BKC-1 from *Klebsiella* has been known to engage both Sec and Tat translocons in that organism [178], whereas the export route of *Shinella* BKC-1 has not been elucidated in the native organism.

Pre-β-lactamases are directed to the secretory pathways based on their N-terminal tripartite signal sequences (Fig. 5) [175]. The signal sequence also determines whether, after its translocation, the mature protein will be free in the periplasmic/extra-cytoplasmic space, or lipidated at the cytoplasmic/outer membrane and consequently membrane anchored [123]. The signal sequence is a short peptide that is superficially similar between the Sec and Tat pathways and is composed of n-, h-, and c-regions. The latter usually contains a recognition sequence for a signal peptidase enzyme, which cleaves the signal peptide during the translocation process [181]. The Sec system signal sequence is characterised by the presence of a basic n-region, a hydrophobic h-region and a polar c-region. In comparison, Tat targeting sequences have some key differences [182]. Most importantly they contain a twin arginine motif in the n-region, critical for interaction with the Tat machinery; conservative substitution of even one of these two arginine amino acids with lysine is sufficient to severely affect Tat transport [183]. While Sec signal peptides also have one or a pair of basic residues in their n-region, lysine, rather than arginine, is favoured [184]. Furthermore, the h-region of the Tat signal is only moderately hydrophobic, and the c-region often has one or more basic residue prior to the signal peptide cleavage site [182]. The co-occurrence of the Sec and Tat pathways leads to a significant evolutionary pressure on the signal peptides to prevent mis-targeting of substrates. For example, increased hydrophobicity of the Tat h-region does not prevent recognition by the Tat translocon but can lead to simultaneous recognition by the Sec [185]. This immediately becomes problematic because folded substrates jam the Sec channel and can be lethal for bacteria [186]. Despite these inherent differences, some signal peptides can be promiscuous and successfully target passenger proteins to both pathways, as observed in the class A carbapenemase BKC-1 [187] that is discussed in more detail below.

### Translocation via the Sec system

The Sec translocon is highly conserved and in bacteria comprises the SecYEG membrane proteins, with SecY forming the central protein-conducting channel [188], and the SecDF accessory complex enhancing translocation efficiency [189]. Sec-mediated protein translocation occurs in three stages: [1] sorting and targeting of the pre-protein to the translocon, [2] translocation of the unfolded peptide chain through SecY into the extra-cytoplasmic space (or insertion into the cytoplasmic membrane for Sec-dependent membrane proteins), and [3] cleavage of the signal sequence and release of the mature protein.

Substrates can be targeted to the Sec system either co-translationally or post-translationally. Most periplasmic and outer membrane proteins are translocated post-translationally, including pre-β-lactamases [173]. Such substrates have targeting signals that are less hydrophobic compared to those of proteins going through the co-translational route [190], and are delivered to the translocase motor SecA, either in the cytoplasm or when it is already docked on the SecYEG complex [191]. Some research also suggests that targeting of pre-TEM to the Sec translocon may be mediated by a direct interaction between the ribosome and the SecA protein, although translocation still occurs post-translationally [191]. Insertion of the signal peptide or signal anchor sequence into the SecY channel unlocks the translocon for protein passage and the pre-β-lactamase is then shuttled across the membrane using ATP hydrolysis and the proton-motive force (PMF) [192]. During the transport process, membrane-anchored signal peptidases SPaseI or SpaseII [175, 193] recognize a consensus site in the c-region of the signal peptide that allows for its proteolytic removal (Fig. 5). Other components linked to the Sec translocon include the dedicated chaperone SecB [194] and the signal recognition particle (SRP) [195], neither of which play a role in pre-β-lactamase translocation, as shown using TEM-1 [125, 129, 153].

Translocation via the Sec pathway has been observed for commonly reported enzymes, such as TEM-1, CTX-M-14 and AmpC from Enterobacteriaceae, L1 from *S. maltophilia* [177], and most recently for the carbapenemase KPC-2 from *K. pneumoniae* [178]. Notably, all metallo-β-lactamase enzymes studied to date, undergo Sec transport, a step critical for selective acquisition of Zn(II) ions in the periplasm [82], and it is thus likely that all enzymes of this family are Sec substrates. Although post-translational (SecA-dependent) and co-translational (SRP-dependent) targeting pathways are generally independent of one another, mutating signal peptides of post-translationally targeted proteins to increase their hydrophobicity can re-route them to the SRP pathway [154, 190]. On that front, and despite being a post-translational substrate of the Sec pathway, TEM-1 can also be compatible with co-translational export, and indeed has long been used in that capacity, as a reporter for *E. coli* membrane protein topology studies [196].

### Translocation via the Tat Pathway

The Tat pathway operates in parallel to Sec in the cytoplasmic membranes of bacteria and archaea. Its defining feature is that it transports folded proteins, and even protein complexes [174], and overall, has a more limited set of substrates than the Sec pathway. In some bacteria, for example *Lactococcus *, it is completely absent [197]. Nonetheless, in many key pathogens it plays an important role in several cellular processes including respiration [198], cell division [199, 200], iron acquisition [201], and virulence [202].

In Gram-negative bacteria such as *E. coli *, the components of the Tat machinery are the TatA, TatB and TatC membrane proteins [174], while in many Gram-positive bacteria, the system is simpler, with only TatA and TatC subunits [174]. In *E. coli *, a complex of TatABC acts as the Tat receptor, with recognition of the twin arginine residues of the Tat signal peptide carried out by TatC [203]. Interaction of the signal peptide with this complex triggers its reorganisation and the binding of further copies of TatA to form a channel, or a patch that weakens the phospholipid bilayer [182]. Unlike the Sec pathway, where both ATP hydrolysis and the PMF drive protein translocation, Tat transport ensues TatA multimerization powered solely by the PMF [204]. Following translocation, the substrate signal peptide is cleaved by either SPase I or SPase II, in a similar manner as for Sec signal peptides [193, 205]. Relatively few β-lactamases have been shown to use the Tat pathway for export. Those that have been described in pathogenic organism include L2 from *S. maltophilia* [177], BlaC from *M. tuberculosis* [179], BRO-2 from *Moraxella catarrhalis* [206], BlaA from *Yersinia enterocolitica* [207], and PenA from *Burkholderia pseudomallei* [180]. Interestingly, the normally Sec-dependent TEM-1 β-lactamase can be efficiently exported in an active form by the Tat pathway if it is provided with a twin arginine signal peptide, and has even been successfully used as a Tat reporter protein [177, 208].

### Overlaps in translocation

Correct targeting of substrates to each of these translocation pathways is essential to ensure that proteins are not irreversibly trapped in the cytoplasm and to avoid lethal jamming of the Sec translocon. Although translocation of Sec and Tat substrates is generally mutually exclusive, signal peptides do show some level of promiscuity. Indeed, BKC-1 [209], a plasmid-encoded β-lactamase thought to have originated from a *Shinella* spp. [210] was recently found to utilise both the Sec and Tat pathways for optimum resistance in *E. coli* and *K. pneumoniae* [178]. This dual-targeted translocation was attributed to the presence of a twin arginine motif in addition to a duplicated sequence of 16, predominantly hydrophobic, amino acids at its N-terminus (Fig. 5); these may have evolved in *K. pneumoniae* upon β-lactam pressure, such as ceftazidime treatment. Another explanation could be that high expression of BKC-1 might lead to the utilisation of the Tat pathway in order to avoid aggregation in the cytoplasm, as observed for LipA in *Bacillus subtilis* [211].

### Signal peptide cleavage

The signal peptides of most periplasmic proteins in Gram-negative bacteria are cleaved by SPase I, which often recognizes a motif in the c-region commonly represented as Ala-Xxx-Ala, whereby Xxx can be any amino acid (but often is a bulky and hydrophobic residue) and the first Ala may also be another small-chain amino acid, such as Gly [175, 193, 212, 213]. By contrast, SPase II recognises the signal peptide c-regions of pre-lipoproteins which contain the Leu-(Ala/Ser/Tyr)-(Gly/Ala) motif and an invariant cysteine at the +1 position of the mature protein, in the sequence area, known as the lipobox; this cysteine is fatty acylated prior to cleavage leading to anchoring of the mature protein to the outer leaflet of the cytoplasmic membrane or into the outer membrane (this is discussed further in the next section) [193, 205]. Cytoplasmic membrane-anchored lipoproteins are particularly abundant in Gram-positive bacteria [214] and include the Sec-dependent PC1 penicillinase found in *S. aureus* [39, 215, 216] and BcIII/BlaP from *Bacillus* spp. [145, 217–220]. Though less common, some Gram-negative species also carry membrane-anchored β-lactamases, including NDM-1 [176, 221], PenA [180] and BRO-2 [206] (Fig. 5).

## Post-translocation: the transformation into an active enzyme

**Fig. 6.**
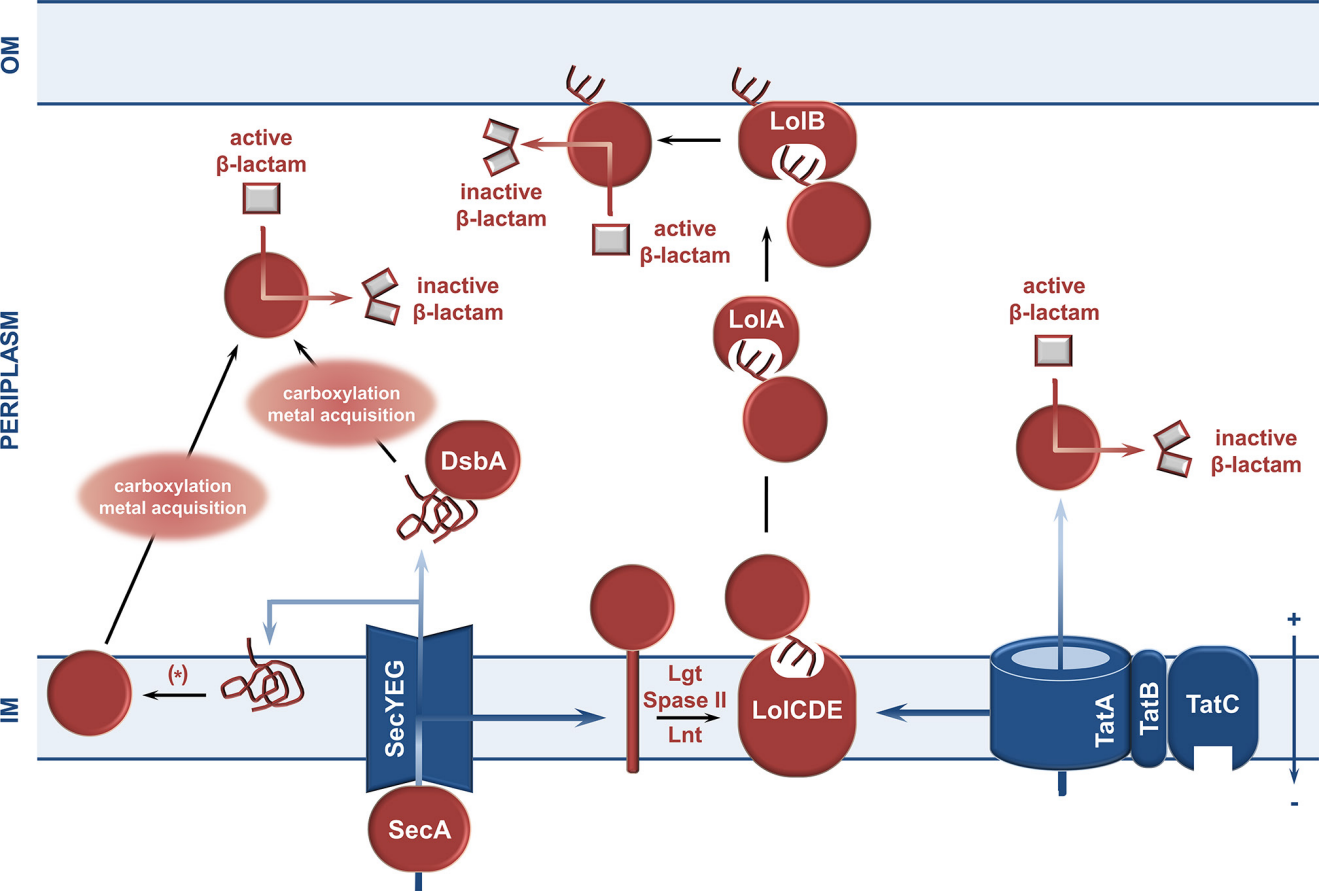
Overview of the post-translocation trajectory of β-lactamases. Sec-translocated β-lactamase enzymes (left) can remain transiently associated with the cytoplasmic membrane and, after folding into their native structures, they are released as soluble enzymes into the extra-cytoplasmic space. The folding of most, enzymes containing two or more cysteine residues depends on the disulfide bond formation pathway, and in particular on the thiol-disulfide oxidase DsbA. *These two processes are not mutually exclusive, as release from the membrane has been proposed to depend on disulfide bond formation [228]. Carboxylation of specific active-site residues or metal acquisition (especially incorporation of Zn(II)), also occur after enzyme translocation and facilitate protein folding. There is no evidence that Tat-transported enzymes (right) require additional folding steps after they reach the extra-cytoplasmic environment. Membrane-anchored enzymes, exported through either the Sec or the Tat systems, are recognised by their ‘lipobox’ during translocation, and are first lipidated, and then transferred to the outer membrane by the Lol system (middle). OM, outer membrane; IM, inner/cytoplasmic membrane.

Following β-lactamase translocation, a rapid, spontaneous, and energetically favourable folding process allows the transported enzyme to adopt the lowest energy conformation, ensuring that it will be soluble in the aqueous extra-cytoplasmic space. The assumption of the final tertiary structure of the β-lactamase occurs through the formation of units of secondary structure, such as α-helices, β-sheets, and, less commonly, β- or Ω-loops [222], followed by condensation to the final folded protein, a catalytically active enzyme that is ready to migrate to the right cellular location. In Gram-negative bacteria this process occurs in the oxidative environment of the periplasm and is supported by folding catalysts, such as the thiol-disulfide oxidoreductases of the disulfide bond formation (DSB) system, as well as a suite of post-translational modifications (see Fig. 6). By contrast, the absence of a periplasmic compartment in Gram-positive species means that protein maturation must take place in the cytoplasm, the cell membrane or the extra-cellular environment [223].

### Newly translocated β-lactamases remain bound to the outer leaflet of the cytoplasmic membrane

After translocation, β-lactamases may remain bound to the membrane [224, 225] where they form early intermediates [226]. Subsequently, conformational changes in the main body of the protein are proposed to lead to its release from the membrane [227, 228]. Studies performed using TEM-1 have demonstrated that certain mutations, for example truncations at the C-terminus of the enzyme, block its release into the periplasm [127, 226]. Despite remaining associated, membrane-bound TEM-1 is catalytically active and capable of conferring resistance to ampicillin in *E. coli* [229]. Thus, the inability to detach from the membrane does not result in protein misfolding and/or degradation.

The remainder of this section gives a brief overview of post-translocation folding processes for all types of β-lactamase enzymes; these are not presented sequentially or in order of importance as the procession of these events remains uncertain and it is likely that several of them take place at the same time.

### Conserved non-catalytic residues direct β-lactamase folding

In polypeptides, amino acid sequence conservation tends to be minimized, ensuring that proteins have the capacity to evolve [230]. As a result, highly conserved residues are often restricted to the catalytically active site of a protein, while less conserved regions determine substrate specificity [10, 231]. In line with this, and despite major differences at the level of primary amino acid sequence, post-translocation β-lactamase folding results in highly similar structural folds within all phylogenetic classes [10, 231] with only a few conserved amino acids playing a key role in the folding process [63, 230, 232, 233].

Comparison of sequences from class A β-lactamases led to the identification of two catalytically inactive residues that are important for the folding of the broad spectrum β-lactamase BlaC [230, 234]. BlaC variants where Glu37 and Trp229 were replaced, were poorly expressed and showed reduced activity against β-lactams when produced in *E. coli *, suggesting defects in protein folding [230]. The loss of Glu37 in particular, led to complete abrogation of resistance, likely due to defective formation of the central β-sheet in an early folding intermediate [230]. The importance of Glu37 has also been highlighted in the metallo-β-lactamase BcII from *Bacillus cereus *, where its loss led to lower hydrolytic activity [232, 235]. Similarly, the contribution of Trp229 to protein folding has also been noted in TEM-1 [233]. In addition to these residues, the folding of many class A β-lactamases, including TEM-1, is dependent on the successful isomerization of the Glu166-Xxx167 (where Xxx can be any amino acid) bond from a *trans* to a *cis* conformation, a step that has been shown to be rate-limiting for enzyme folding [36]. Appropriate orientation of this bond is critical for the correct positioning of Glu166, which is one of the key active-site residues for hydrolytic activity [36, 236].

In the case of class D β-lactamases, alignment of OXA protein sequences revealed several conserved regions in that enzyme family, and singled out Trp154, a residue located in the Ω-loop [59, 63]. The loss of the Trp154 side chain increases the flexibility of the Ω-loop, which is, in part, responsible for the shape and overall charge of the active site. Mutations in that position result in low protein yields during purification, once again indicating a key role of this residue in enzyme folding [59, 63].

### Formation of disulfide bonds underpins the folding of cysteine-containing β-lactamase enzymes

Most amino acid sequences can spontaneously adopt highly ordered states guided by hydrogen bonding, electrostatic forces and van der Waals interactions. In addition to these, prokaryotic extra-cytoplasmic proteins, like β-lactamases, often contain even numbers of cysteine residues that, almost always, interconnect to form disulfide bonds [223]. These covalent linkages are present in enzymes from all four β-lactamase Ambler classes [7]. Moreover, the position of the disulfide bond is largely conserved within each phylogenetic class of enzymes, indicating that these structural elements could be important for the folding and activity of β-lactamases [7, 59, 237].

Until recently, the role of disulfides for β-lactamase folding remained unclear. Computational analyses of the ESBL OXA-1, had proposed that the disulfide bond alters the dynamics and specificity of the Ω-loop region and, therefore could stabilize the catalytic centre of the enzyme [59]. In agreement with this, introduction of a non-native disulfide in the ESBL TOHO-1, showed increase in its thermal stability [238, 239]. A few additional lines of evidence also hinted that disulfides might be important. For example, removal of the cysteines from enzymes of the GES family results in protein precipitation [237], while the carbapenemase SME requires its cysteines to confer resistance to β-lactam compounds [240].

A recent study has demonstrated that disulfide bond formation is essential for many clinically important β-lactamase enzymes that contain two or more cysteine residues and are translocated into the cell envelope through the Sec system [7]. The activity of several ESBL and carbapenemase enzymes from Class A (KPC, GES, FRI, SME families), B3 (L1 family) and D (OXA family) was tested in *E. coli* lacking the primary oxidase of the DSB system. In the absence of the oxidative protein folding activity of DsbA, *E. coli* strains expressing these enzymes could no longer survive in the presence of complex β-lactams (cephalosporins, carbapenems and monobactams), due to enzyme misfolding and, in most cases, degradation. In the same study, similar results were obtained in clinical isolates of *E. coli *, *K. pneumoniae *, *Enterobacter cloacae *, *C. freundii* and *P. aeruginosa* expressing these enzymes, either by using a DSB system inhibitor or by deleting *dsbA* [7]. Generally, the greatest effects were observed for enzymes with broad hydrolytic activities, whilst narrow-spectrum enzymes, such as SHV, were less dependent on their disulfide bonds [7]. This is in agreement with early observations by Schultz *et al*. where removal of the disulfide bond from the narrow-spectrum enzyme TEM-1 did not affect its activity under physiological conditions, but was, nonetheless, detrimental when temperature or pH stresses were applied [241].

Although the mechanism behind β-lactamase release from the membrane has not been elucidated, research using TEM-1 mutants showed that the cysteine residues of this enzyme are key for membrane release in *Salmonella typhimurium* [228]. Disulfide bond formation plays a central role in the folding process of extra-cytoplasmic proteins [242], and in this case seems to be important for the conformational change required for release of TEM-1 from the membrane into the periplasm.

### Membrane-anchored β-lactamases require lipidation of their signal sequence

Modification of the pre-lipoprotein occurs at the periplasmic leaflet of the inner membrane following translocation by the Sec or the Tat system and recognition of its ‘lipobox’ conserved sequence (Fig. 5) by a three-protein modification pathway (Fig. 6) [243]. The pre-lipoprotein diacylglyceryl transferase (Lgt) catalyses the formation of a thio-ether bond between the invariant +1 cysteine residue in the lipobox and a diacylglycerol group of a phosphatidylglycerol [243]; the signal sequence is then cleaved by Spase II at the newly modified cysteine [244–247]. N-acylation of the cysteine amine group by the apolipoprotein N-acyltransferase (Lnt) [247–249] with a long-chain fatty acid, results in the formation of a mature lipidated membrane anchor sequence; this step is critical for the release of the lipo-β-lactamase to the localization of lipoprotein (Lol) pathway, which transports it and finally embeds it into the outer membrane [123, 218, 243, 250, 251].

### Residue-specific post-translational modifications affect β-lactamase structure and function

In addition to disulfide bond formation and *cis* to *trans* bond isomerisation, other post-translational modifications are sometimes required for β-lactamase enzymes to be active. For example, many class D β-lactamases, such as OXA enzymes, rely on the ability of a carboxylated Lys70 to interact with the catalytic serine residue (Ser67) and the water molecule it coordinates, so as to act as a base during β-lactam hydrolysis [60, 62, 63]. This is achieved by spontaneous carboxylation of the amine side-chain of this residue, resulting in reorganization of the active-site hydrogen bonds and subsequent stabilization of Lys70 by Trp154 [62, 63]; the latter is the same amino acid that has been shown to play a key role in protein folding. Similarly, the equivalent residue, Lys104, is carboxylated in another class D β-lactamase, BPU-1 from *Bacillus* spp. [65]. As this lysine amino acid, which is part of the active site, is commonly conserved, it is likely that this type of modification also occurs in other members of class D enzymes.

### Metal acquisition in class B β-lactamases

Class B metallo-β-lactamases rely on an additional step during folding to achieve full functionality, namely the acquisition of one or two zinc metal ions. Despite some variations in the metal coordination site residues between the class B sub-groups, class B enzymes exhibit strong preference for Zn(II) binding and this specificity appears to be linked to the translocation of these proteins into the periplasm [80, 252]. For example, although GOB-18 binds Zn(II) and Fe(II) when isolated from the cytoplasm, with both forms being functional, only the Zn(II)-binding form has been observed when the same enzyme is expressed in the periplasm [80]. Similarly, the carbapenemase L1 has been shown to be active when containing Fe(II) or Fe(II)/Zn(II) in metal limiting conditions, but Zn(II) is preferentially bound in its active site under standard growth conditions [252].

### Mitigating β-lactamase misfolding

The numerous steps required for the correct folding of β-lactamase enzymes, as well as the chemical and mechanical stresses that extra-cytoplasmic proteins must endure, necessitate the presence of processes responsible for protein quality control, i.e. refolding or degrading any enzymes that fail to adopt their native active fold. In that respect, TEM-1 has been shown to interact with the chaperone/protease DegP under temperature stress conditions. This makes sense since DegP degrades many proteins containing disulfide bonds, especially when the latter fail to form [253], and, for this reason, is likely to interact with other disulfide-containing β-lactamases when they are misfolded or denatured.

## Post-translocation: reaching the final destination

Although serine-β-lactamases have evolved from the membrane-anchored PBPs, they are mostly soluble enzymes localised in the periplasm of Gram-negative bacteria [254–256]. Experiments using native and engineered inner-membrane-anchored TEM-1 suggest that the solubilisation of these hydrolases offers long-term expression and survival advantages [13]. The few existing examples of membrane-embedded enzymes are restricted to species where the secretion of these proteins in a soluble form would be disadvantageous and would result in inefficient use of cellular resources. For example, in Gram-positive bacteria, enzyme secretion would lead to dilution of the protective effects conferred by the β-lactamase, due to its diffusion away from the producing cell. Similarly, embedding of metallo-β-lactamases in the outer membrane or their incorporation in outer membrane vesicles (OMVs) has been proposed to protect them from protease degradation under Zn(II)-limiting conditions [176, 257].

### Membrane-anchored β-lactamases and their secretion

Membrane-anchored β-lactamases are synthesized as pre-lipoproteins and post-translational lipid addition to these enzyme precursors leads to their incorporation into the membrane [219, 258, 259]. In many Gram-negative bacteria, with Enterobacteriaceae being the prototypical example, the Lol pathway is then responsible for the transport of the anchored β-lactamase molecule from the inner to the outer membrane (Fig. 6). The lipoprotein is recognised by LolE [260] and extracted from the inner membrane by the LolCDE complex, a process energized through ATP hydrolysis [261, 262]. The lipoprotein is subsequently solubilised in the periplasm through its interaction with the chaperone LolA, which shields its hydrophobic lipid region [247, 251, 260, 262], and interaction between LolA and LolB leads to transfer of the β-lactamase into the outer membrane [247, 263, 264]. Outer membrane anchoring has been shown for the Tat-translocated PenA enzyme from *B. pseudomallei* [265], as well as the metallo-β-lactamase NDM-1 [176]. Conversely, BRO-1 has been shown to be minimally embedded (10 %) in the outer membrane of its natural host, *M. catarrhalis *, but when expressed in *E. coli *, it inserts into the outer membrane at much higher levels [266, 267]. To date, β-lactamase secretion into the extracellular milieu for Gram-negative species has only been proposed for a class C β-lactamase from *Psychrobacter immobilis* [268]. In Gram-positive bacteria membrane-anchored enzymes localise in the plasma membrane and include examples such as the class A penicillinase from *Streptomyces griseus *, PC1 from *S. aureus* [269], and BcIII/ BlaP from *Bacillus* spp. [145, 217–220 ]. In addition to their membrane attachment, PC1 (144 216), BcIII, and BlaP enzymes have been also detected in the supernatant, whereby they are released after proteolytic cleavage of their lipidated anchor [145, 217, 218, 257].

### β-Lactamase release through incorporation in OMVs

In addition to achieving increased localised concentration, membrane anchoring of β-lactamases also favours the packaging of these proteins into OMVs, therefore allowing their extra-cellular release, which has been linked to improved cell survival under antibiotic stress [176, 254, 255, 270–277]. This phenomenon has been studied in-depth in Gram-negative species, including *P. aeruginosa* [271] and *A. baumannii* [278], as well as specifically for enzymes like NDM-1 [176], and BRO-1 [254, 266]. OMV secretion, in addition to periplasmic expression, of the resident L1 and L2 β-lactamases of *S. maltophilia* has also been proposed and is thought to occur in a dose-dependent manner following imipenem exposure [276]. Finally, several clinical isolates of *P. aeruginosa *, have been shown to incorporate a class C β-lactamase in their OMVs [271], while the same has been observed for β-lactamases from *S. aureus* [272, 279, 280].

Effective packaging of β-lactamases into OMVs has been attributed to enzyme-specific electrostatic interactions with the membrane. In particular, a recent study in *E. coli* demonstrated interactions between anionic lipids of the outer membrane and the conserved basic residues Arg45 and Arg52 in NDM-1, or lysine residues in IMP-1 [281]. Further confirming this observation, introduction of residues promoting such interactions into the soluble metallo-β-lactamase VIM-1 also led to its incorporation into OMVs [281]. While insertion of NDM-1 into bacterial membranes and OMVs does not impose a noticeable burden on the producing organism [281], the expression of species-specific β-lactamases such as SPM-1 (* P. aeruginosa *) induces envelope stress and hypervesiculation in non-native producers [122, 281]. This suggests that secretion through OMVs could impose a burden in cases where such enzymes are mobilized into new hosts. In addition, OMV secretion can enhance ‘cheater’ emergence, since OMV-secreted β-lactamases have been shown to protect nearby non-producing bacterial populations from otherwise lethal antibiotic stress [176]. It should be noted that in most cases it is unclear whether β-lactamase release via OMVs is ‘intentional’ or merely a by-product of the large quantities of these enzymes translocated into the periplasm.

### β-Lactamase secretion via the ype I secretion system

Last but not least, β-lactamase secretion has recently been linked to the Type I (T1SS) secretion system, since the activity of CTX-M-15 from an environmentally isolated multidrug resistant *E. coli* was shown to be partially dependent on the T1SS component TolC [282]. It remains unclear as to how TolC mediates this process, and whether this could be a general secretion mechanism also utilised by other β-lactamase enzymes.

## Concluding remarks

The activity of β-lactamases is dependent on their correct folding and localisation outside the cytoplasmic compartment of bacterial cells. While this has been long known, the biogenesis of these enzymes has rarely been explicitly studied. More commonly, and due to the fact that their activity can be easily measured, β-lactamases have been used as reporting tools in experiments primarily aiming to investigate the function of bacterial chaperones or translocation systems. These experiments, mostly performed in the 90s or the early 2000s, form a big part of the knowledge base around the biogenesis of β-lactamases presented in this review. More recent studies, specifically focusing on the translocation or folding of these resistance determinants, have shown that for some β-lactamases, their hydrolytic spectrum depends on their export route [178] or that oxidative folding processes are essential for their activity [7]. These works highlight how an in-depth understanding of the biogenesis process of β-lactamases might prove important as the basis for the development of entirely novel next-generation strategies aiming to abrogate the function of these enzymes, thus potentiating existing invaluable antibiotics.
